# Supplementary material for: Early-life disruption of amphibian microbiota decreases later-life resistance to parasites
Source: Nat Commun. 2017 Jul 20;8:86. doi: 10.1038/s41467-017-00119-0 (PMC5519754; doi:10.1038/s41467-017-00119-0)
Supplement: Supplementary file 1 — Supplementary Information [file 41467_2017_119_MOESM1_ESM.pdf]

File name: Supplementary Information
Description: Supplementary Figures and Supplementary Tables

File name: Peer Review File
Description:

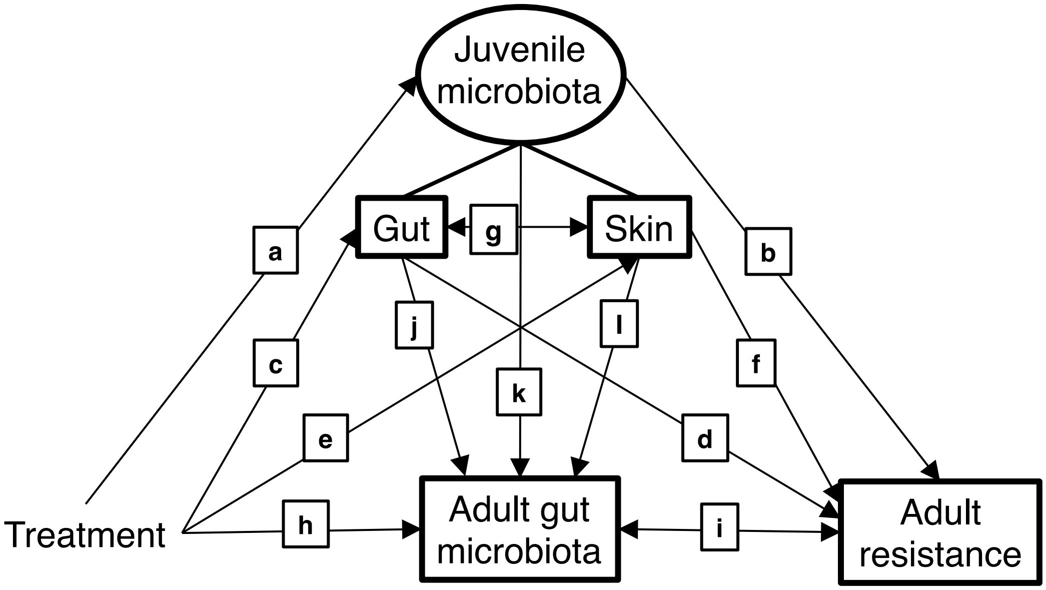

Supplementary Figure 1: Full SEM model. Full model depicts the relationships among the juvenile water treatment, juvenile bacterial diversity (including gut and skin bacteria), adult gut bacterial diversity, and adult resistance to infections. Each letter indicates a path that was tested in one of the eight SEMs models that we competed.

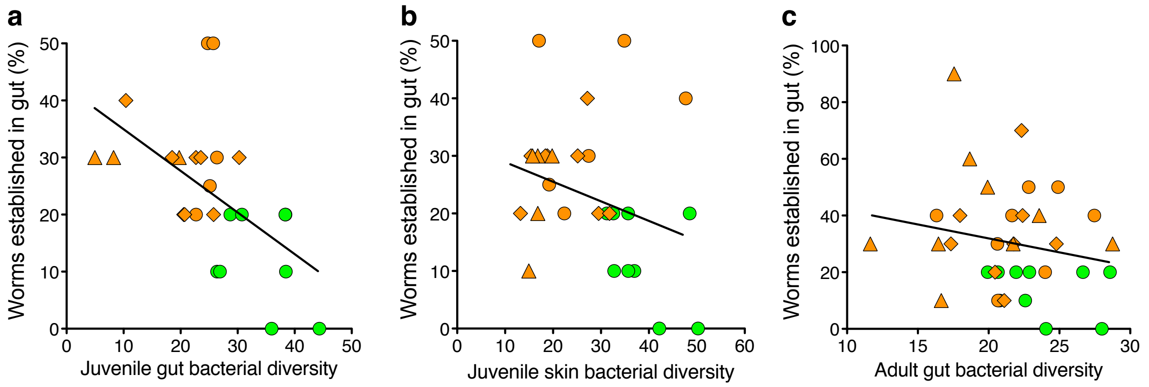

Supplementary Figure 2: Relationship between bacterial phylogenetic diversity and the percent of worms that established in the guts of adult frogs for: (**a**) juvenile guts, (**b**) juvenile skin, and (**c**) adult guts. Bacterial diversity of the guts of juveniles significantly predicted infection risk (n = 25 tanks; GLM, χ^2 = 6.28, df = 1, P = 0.01), but bacterial diversity for juvenile skin (n = 28 tanks; χ^2 = 2.87, df = 1, P = 0.09) and adult guts (n = 35 tanks; GLMM, χ^2 = 2.54, df = 1, P = 0.11) was not related significantly to infection risk. Bacterial diversity from juveniles and adults also did not significantly predict the number of worms that penetrated the host (juvenile gut: χ^2 = 0.47, df = 1, P = 0.49, juvenile skin: χ^2 = 3.38, df = 1, P = 0.07; adult gut: (χ^2 = 0.18, df = 1, P = 0.67). Different shapes and shading represent the different water treatments: pond water (green circles), sterile pond water only (orange circles), sterile pond water and short-term antibiotic water (orange diamonds), sterile pond water and long-term antibiotic water (orange triangles). Juvenile and adult samples were collected from different individuals within the same replicate (tank) because tadpole sampling required destructive sampling. Juvenile and adult samples from the same tank were then paired for analyses.

Supplementary Table 1: Mean (±s.e.m.) phylogenetic diversity (PD), Shannon index (SI), species richness (SR), and species evenness (SEV) for frogs reared in pond water (PW), sterile pond water only (SPW), sterile pond water and short-term antibiotics (STAB), or sterile pond water and long-term antibiotics (LTAB). Numbers in parentheses represent the number of replicates. Statistics represent the outcomes of GLMs for juveniles or GLMMs for adults (with tank as a random effect). Treatments that do not share letters are significantly different based on a sequential Bonferroni post-hoc multiple comparison test (*P* < 0.05).

Diversity index	PW	SPW	STAB	LTAB	Statistics*
Juvenile Guts					
PD	34.25 ± 1.87^{a} (10)	26.12 ± 2.47^{b} (9)	21.77 ± 1.82^{b} (9)	10.35 ± 2.47^{c} (8)	$\chi^2 = 62.99$, $P < 0.0001$
SI	6.63 ± 0.60^{a} (10)	$5.93 \pm 0.28^{a,b}$ (9)	4.97 ± 0.45^{b} (9)	2.83 ± 0.71^{c} (8)	$\chi^2 = 43.49$, $P < 0.0001$
SR	334.2 ± 19.2^{a} (10)	263.2 ± 21.2^{b} (9)	208.5 ± 23.0^{b} (9)	69.2 ± 21.8^{c} (8)	$\chi^2 = 80.76$, $P < 0.0001$
SEV	0.79 ± 0.02^{a} (10)	$0.74 \pm 0.03^{a,b}$ (9)	0.64 ± 0.05^{b} (9)	0.46 ± 0.09^{c} (8)	$\chi^2 = 28.05$, $P < 0.0001$
Juvenile Skin					
PD	37.41 ± 2.32^{a} (10)	27.13 ± 2.80^{b} (10)	$22.43 \pm 2.43^{b,c}$ (8)	$16.66 \pm 0.64^{c,d}$ (10)	$\chi^2 = 49.86$, $P = < 0.0001$
SI	6.70 ± 0.18^{a} (10)	5.63 ± 0.33^{b} (10)	5.15 ± 0.50^{b} (8)	5.21 ± 0.17^{b} (10)	$\chi^2 = 17.37$, $P < 0.001$
SR	441.0 ± 21.9^{a} (10)	313.7 ± 24.8^{b} (10)	278.0 ± 30.4^{b} (8)	207.4 ± 12.6^{c} (10)	$\chi^2 = 58.98$, $P < 0.0001$
SEV	0.76 ± 0.02^{a} (10)	$0.68 \pm 0.03^{a,b}$ (10)	0.63 ± 0.05^{b} (8)	$0.68 \pm 0.02^{a,b}$ (10)	$\chi^2 = 9.69$, $P = 0.02$
Adult Guts					
PD	22.68 ± 0.50^{a} (10)	23.11 ± 1.17^{a} (10)	22.35 ± 0.75^{a} (10)	18.90 ± 0.95^{a} (11)	$\chi^2 = 13.86$, $P = 0.003$
SI	5.03 ± 0.13^{a} (10)	5.15 ± 0.21^{a} (10)	4.95 ± 0.14^{a} (10)	4.49 ± 0.19^{b} (11)	$\chi^2 = 9.04$, $P = 0.03$
SR	189.8 ± 5.5^{a} (10)	192.8 ± 13.5^{a} (10)	185.0 ± 9.34^{a} (10)	160.2 ± 9.62^{a} (11)	$\chi^2 = 6.33$, $P = 0.10$
SEV	0.67 ± 0.01^{a} (10)	0.68 ± 0.02^{a} (10)	$0.66 \pm 0.01^{a,b}$ (10)	0.61 ± 0.02^{b} (11)	$\chi^2 = 8.46$, $P = 0.04$

*df = 3 for all χ^2

Supplementary Table 2: Comparison of the eight hypothesized *a priori* models for the relationships among treatment, juvenile bacterial diversity (gut and skin), adult gut bacterial diversity, and adult resistance to infections. The models were ranked by Akaike information criterion (AIC). See Supplemental Figure 1 for the paths that correspond to each letter in each model.

**Model**	**Paths**	**AIC**	**ΔAIC**	**ω**
1	a-b	333.15	0.00	0.92
2	c-f	337.97	4.82	0.08
3	a-b, i	409.87	76.72	0.00
4	a-b, h-i	415.21	82.06	0.00
5	a-b, h-i, k	422.59	89.44	0.00
6	a-b, i, k	425.21	92.06	0.00
7	a, k, i	425.21	92.06	0.00
8	c-f, h-j, l	436.91	103.76	0.00

Supplementary Table 3: The sub-lethal and lethal effects of juvenile water treatment (pond water (PW), sterile pond water only (SPW), sterile pond water and short-term antibiotics (STAB), or sterile pond water and long-term antibiotics (LTAB)) on juvenile and adult frogs. Days to metamorphosis and mass are represented by mean (±s.e.m.) days and grams, respectively. For juvenile and adult survival, the numbers represent the number of individuals over the number of tanks. Numbers in parentheses represent the number of replicates. See the main text for the results of the statistical analyses.

**Treatment**	**Days to metamorphosis**	**Mass at metamorphosis**	**Juvenile survival**	**Adult mass**	**Adult survival**
PW	53.45 ± 2.68 (19)	0.40 ± 0.03 (15)	51/19	0.68 ± 0.06 (16)	34/16
SPW	50.88 ± 3.49 (20)	0.45 ± 0.03 (20)	59/20	0.76 ± 0.04 (20)	40/20
STAB	45.81 ± 3.45 (20)	0.38 ± 0.02 (19)	59/20	0.74 ± 0.05 (19)	45/19
LTAB	100.80 ± 4.68 (19)	0.41 ± 0.02 (18)	45/19	0.53 ± 0.02 (19)	31/19

Supplementary Table 4: Ranked models (same methods and pathways as the original SEM) excluding samples from the long-term antibiotic treatment, which reduced growth and survival of frogs. Even without the long-term antibiotic treatment, the top-ranked models remain the same as the original SEM.

**Model**	**Paths**	**AIC**	**ΔAIC**	**ω**
1	a-b	289.57	0.00	0.88
2	c-f	293.57	3.99	0.12
3	a-b, i	350.03	60.45	0.00
6	a-b, i, k	357.36	67.78	0.00
4	a-b, h-i	357.39	67.81	0.00
7	a, k, i	364.95	75.37	0.00
5	a-b, h-i, k	367.71	78.31	0.00
8	c-f, h-j, l	381.08	91.51	0.00

Supplementary Table 5: Ranked models (same methods and pathways as the original SEM) including samples from the long-term antibiotic treatment but also two additional models with adult mass and overall tank survival, which were both lower in frogs from the long-term antibiotic treatment. Models 9 and 10 include the same pathways as models 1 and 2, respectively, from the original analysis but also the effect of adult mass and survival on adult resistance. The top ranked model is the same as the original SEM.

Model	Paths	AIC	ΔAIC	ω
1	a-b	333.15	0.00	0.72
9	a-b + mass and survival	335.79	2.64	0.19
2	c-f	337.97	4.82	0.07
10	c-f + mass and survival	340.75	7.60	0.02
3	a-b, i	409.97	76.72	0.00
6	a-b, i, k	415.13	81.98	0.00
4	a-b, h-i	415.21	82.06	0.00
5	a-b, h-i, k	422.59	89.44	0.00
7	a, k, i	425.21	92.06	0.00
8	c-f, h-j, l	436.91	103.76	0.00

Supplementary Table 6: Significant relationships between the relative abundance of bacterial genera in juveniles and adults and the percent of worms found in the guts of adult frogs. The directions of the relationships are indicated as positive (+) or negative (-). df = 1 for all χ^2.

**Bacterial genera**	**Juvenile gut**		**Juvenile skin**		**Adult gut**	
	+/-	χ^2, ***P*-value**	+/-	χ^2, ***P*-value**	+/-	χ^2, ***P*-value**
Blastomonas			-	χ^2= 10.80, P = 0.001		
Burkholderia	-	χ^2= 4.63, P = 0.03				
Cetobacterium	-	χ^2= 4.99, P = 0.03	-	χ^2= 7.44, P = 0.006		
Chromobacterium			-	χ^2= 8.13, P = 0.004		
Chryseobacterium			-	χ^2= 5.93, P = 0.01		
Citrobacter					-	χ^2= 8.07, P = 0.005
Coprobacillus			-	χ^2= 5.19, P = 0.02		
Curvibacter			-	χ^2= 14.34, P < 0.001		
Dorea	-	χ^2= 6.27, P = 0.01				
Erwinia	-	χ^2= 7.66, P = 0.006				
Gloeobacter			-	χ^2= 9.48, P = 0.002		
Janthinobacterium					-	χ^2= 7.11, P = 0.008
Morganella					-	χ^2= 5.22, P = 0.02
Ochrobactrum	-	χ^2= 8.77, P = 0.003				
Parabacteroides	-	χ^2= 6.27, P = 0.01				
Perlucidibaca			-	χ^2= 4.10, P = 0.02		
Sediminibacterium			-	χ^2= 14.56, P < 0.001		
Sphingomonas	-	χ^2= 9.77, P = 0.002	-	χ^2= 3.91, P = 0.05		
Tolumonas			+	χ^2= 4.33, P = 0.04		
Treponema			+	χ^2= 9.50, P = 0.002		
u114	-	χ^2= 4.19, P = 0.04	-	χ^2= 5.41, P = 0.02		
